# A U-Net Approach to Apical Lesion Segmentation on Panoramic Radiographs

**DOI:** 10.1155/2022/7035367

**Published:** 2022-01-15

**Authors:** Ibrahim S. Bayrakdar, Kaan Orhan, Özer Çelik, Elif Bilgir, Hande Sağlam, Fatma Akkoca Kaplan, Sinem Atay Görür, Alper Odabaş, Ahmet Faruk Aslan, Ingrid Różyło-Kalinowska

**Affiliations:** ^1^Department of Oral and Maxillofacial Radiology, Faculty of Dentistry, Eskisehir Osmangazi University, Eskisehir 26040, Turkey; ^2^Department of Oral and Maxillofacial Radiology, Faculty of Dentistry, Ankara University, Ankara 06560, Turkey; ^3^Ankara University Medical Design Application and Research Center (MEDITAM), Ankara 06560, Turkey; ^4^Department of Mathematics and Computer Science, Faculty of Science, Eskisehir Osmangazi University, Eskisehir 26040, Turkey; ^5^Department of Dental and Maxillofacial Radiodiagnostics, Medical University of Lublin, Lublin 20-093, Poland

## Abstract

The purpose of the paper was the assessment of the success of an artificial intelligence (AI) algorithm formed on a deep-convolutional neural network (D-CNN) model for the segmentation of apical lesions on dental panoramic radiographs. A total of 470 anonymized panoramic radiographs were used to progress the D-CNN AI model based on the U-Net algorithm (CranioCatch, Eskisehir, Turkey) for the segmentation of apical lesions. The radiographs were obtained from the Radiology Archive of the Department of Oral and Maxillofacial Radiology of the Faculty of Dentistry of Eskisehir Osmangazi University. A U-Net implemented with PyTorch model (version 1.4.0) was used for the segmentation of apical lesions. In the test data set, the AI model segmented 63 periapical lesions on 47 panoramic radiographs. The sensitivity, precision, and F1-score for segmentation of periapical lesions at 70% IoU values were 0.92, 0.84, and 0.88, respectively. AI systems have the potential to overcome clinical problems. AI may facilitate the assessment of periapical pathology based on panoramic radiographs.

## 1. Introduction

Chronic apical periodontitis is an infection of tissues surrounding the dental apex induced by pulpal disease, mostly because of bacterial disease in the root canal complex developing during untreated or incorrectly treated dental caries [1–3]. Apical periodontitis is common, and its prevalence increases with age. Epidemiological studies have reported that apical periodontitis is present in 7% of teeth and 70% of the general population. The diagnosis of acute apical periodontitis is made clinically, but the detection of chronic apical periodontitis is done by radiography [4]. In general, following root canal treatment, complete healing of periapical lesions is expected or at least improvement in the form of a decrease of the size of periapical lesion [1, 5]. Radiographically, apical periodontitis manifests as a widened periodontal ligament space or visible lesions. Such radiolucencies, also called apical lesions, tend to be detected incidentally or by radiographic follow-up of endodontically treated teeth [6, 7]. Radiolucency in radiographs is an important feature of apical periodontitis [2]. Apical periodontitis can be detected on periapical and panoramic radiographs and by cone-beam computed tomography (CBCT). CBCT has superior discriminatory power but is costly and exposes the patient to radiation burden [6, 8]. Periapical and panoramic radiographs are the most frequently used techniques in the diagnosis and treatment of apical lesions [2]. Panoramic radiography generates two-dimensional (2D) tomographic images of the entire maxillomandibular area [9], enabling the evaluation of all teeth simultaneously. Also, panoramic radiography requires a far lower dose of radiation than CBCT imaging [6, 10]. Besides, panoramic radiography is painless, unlike intraoral radiographs, thus well tolerated by patients [9, 11]. One of the many recent technological advances in artificial intelligence (AI) and its applications are expanding rapidly, also in the area of medical management and medical imaging [12]. AI uses computational networks (neural networks (NNs)) that mimic biological nervous systems [13]. NNs were developed as one of the first types of AI algorithms. The computing power of NNs varies depending on the character and amount of training data. Networks using many large layers are termed deep learning NNs [14]. A deep convolutional neural network (D-CNN) was used to process large and complex images [15]. Deep learning networks, including CNNs, have displayed superior achievement in terms of object, face, and activity recognition [16]. Medical organ and lesion segmentation are an important application of imaging modalities [17, 18]. The detection and classification performance of deep learning-based CNNs concerning retinopathy caused by diabetes, skin cancer, and tuberculosis is very high [19, 20]. CNNs have also been applied in dentistry for tooth detection and numbering, as well as an assessment of periodontal bone loss and periapical pathology [21–25]. U-Net and pixel-based image segmentation, which is a different architecture created from CNN layers, are more successful than classical models even if there are few training images. The presentation of this architecture has been realized with biomedical images. The traditional U-Net architecture, extended to handle volumetric input, has two phases: the coder portion of the network where it learns representational features at unlikely scale- and gather-dependent information, and the decoder portion where the network extracts knowledge from the noticed situation and formerly learned features. The jump links used between the corresponding encoder and decoder layers allow deep parts of the network to be trained efficiently and compare the same receiver characteristics with different receiver areas [26].

The study is aimed at assessing the diagnostic success of U-Net approach for the segmentation of apical lesions in panoramic images.

## 2. Material and Methods

### 2.1. Radiographic Data Preparation

The panoramic radiographs used in the study were derived from the archives of the Faculty of Dentistry of Eskisehir Osmangazi University; 470 anonymized panoramic radiographs were applied. The radiographs were obtained from January 2018 to January 2019 for a variety of reasons. Images with artifacts of any type were excluded. The study design was authorized by the Non-Interventional Clinical Research Ethics Committee of Eskisehir Osmangazi University (decision date and number: 06.08.2019/14). The study was conducted following the regulations of the Declaration of Helsinki. The Planmeca Promax 2D (Planmeca, Helsinki, Finland) panoramic imaging system was used to obtain panoramic radiographs with the following parameters: 68 kVp, 16 mA, and 13 s.

### 2.2. Image Annotation

Three dental radiologists (I.S.B. and E. B. with 10 years of experience and F.A.K. with 3 years of experience) annotated ground truth images with the common decision on all images using CranioCatch Annotation software (Eskisehir, Turkey). The polygonal boxes were used to determine the locations of the apical lesions.

### 2.3. Deep CNN Architecture

**Figure 1:**
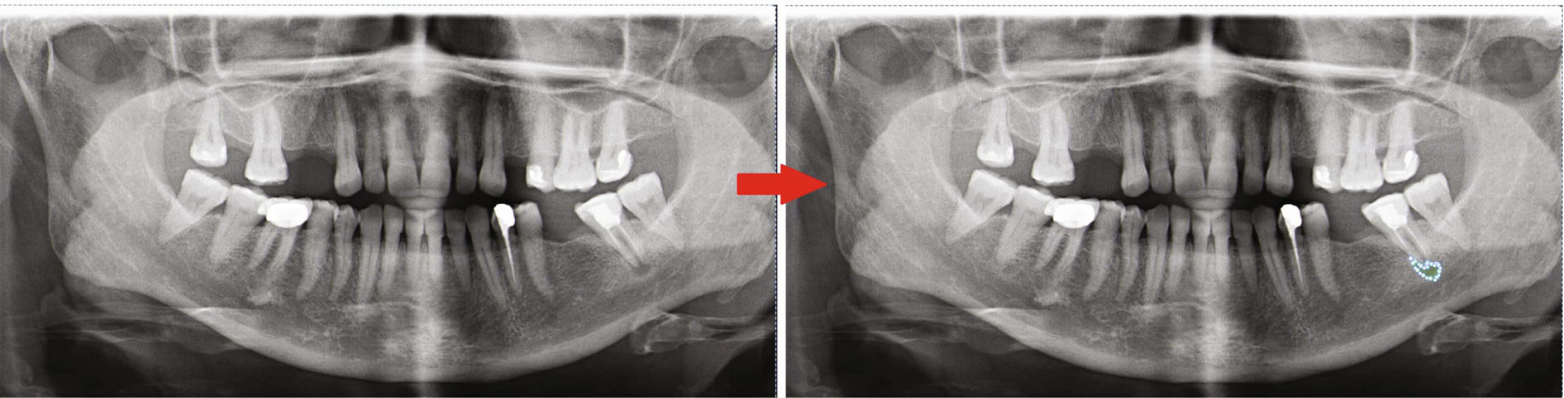
Annotation of the apical lesion using polygonal box method.

The deep learning was performed using a U-Net implemented with the PyTorch model (version 1.4.0). The U-Net architecture is used for semantic segmentation assignments (Figure 1).

The U-Net architecture consists of four block levels, including two convolutional layers with batch normalization and a rectified linear unit activation function (ReLu). There is a maximum pool layer in the encoding section and upconvolution layers in the decoding section. Each block has 32, 64, 128, or 256 convolutional filters. Besides the bottleneck, the layer comprises 512 convolutional filters. Skip connections to the corresponding layers from the encoding layers are present in the decoding part [26]. The Adam Optimizer was used to train the U-Net.

### 2.4. Model Pipeline

PyTorch library was used for model development on the Python open-source programming language (v. 3.6.1; Python Software Foundation, Wilmington, DE, USA; retrieved on August 1, 2019, from https://www.python.org/). An AI model (CranioCatch, Eskisehir-Turkey) was developed to automatically segment apical lesions on panoramic radiographs. The training process was performed using an individual computer implemented with 16 GB RAM and an NVIDIA GeForce GTX 1060Ti graphic card.

- Split: 470 panoramic radiographs were divided into train, validation, and test group

- Training group: 380
- Validation group: 43
- Test group: 47

(ii) Augmentation: 1140 images from the 380 original training group images were derived using data augmentation. Augmentation was applied on the training data set, and augmentations were horizontal flip and vertical flip (total images: 1140 (=380 × 3)) (size: 2943 × 1435)
(iii) Cropping (preprocessing step): then, all images of the train were divided into 4 parts as upper right, upper left, lower right, and lower left (size: 1000 × 530)

- Training group: 1140 × 4 = 4560
- Validation group: 43 × 4 = 172
- Test group: 47

(iv) Remove full black masks (preprocessing step): the regions without lesions of all data set were deleted

- Training group: 1629
- Validation group: 59
- Test group: 47

(v) Contrast Limited Adaptive Histogram Equalization (CLAHE) (preprocessing step): CLAHE has applied all images to improve image contrast and enable the identification of apical lesions

- Training group: 1629
- Validation group: 59
- Test group: 47

(vi) Resize (preprocessing step): the resolution of each piece divided into 4 (1000 × 530) was resized to 512 *x* 256

- Training group: 1629
- Validation group: 59
- Test group: 47

**Figure 2:**
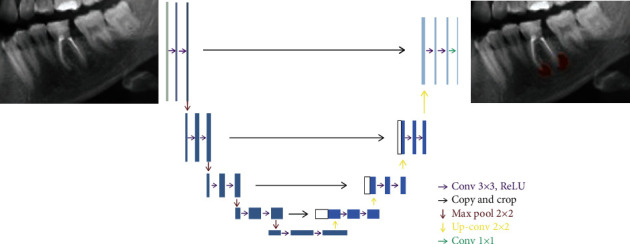
The U-Net architecture for the semantic segmentation task.

The segmentation model with PyTorch U-Net was trained with 95 epochs; the model based on 43 epochs showed the best performance and was thus used in the experiment. The model pipeline is summarized in Figure 2.

### 2.5. Statistical Analysis

The confusion matrix was used to assess the achievement of the model. This matrix is a meaningful table that summarizes the predicted and actual situations. The performance of model is frequently assessed using the data in the confusion matrix [27]. The metrics used to evaluate the success of the model were as follows:

1. True Positive (TP): apical lesion was segmented, correctly
2. False Positive (FP): apical lesions were not detected
3. False Negative (FN): without apical lesions, lesions were nevertheless segmented
4. TP, FP, and FN were determined; then, the following metrics were computed:

- Sensitivity (recall): TP/(TP + FN)
- Precision: TP/(TP + FP)
- F1 score: 2TP/(2TP + FP + FN)

## 3. Results and Discussion

### 3.1. Results

**Figure 3:**
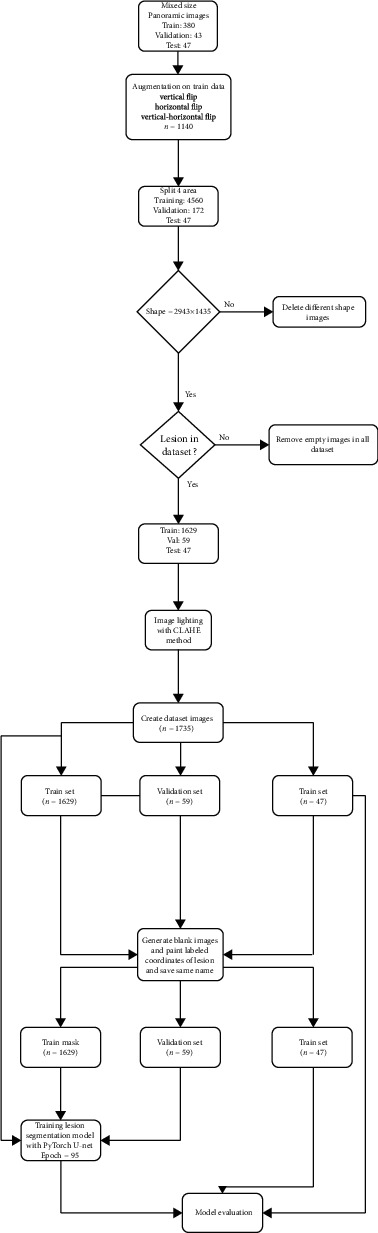
Model pipeline for apical lesion segmentation (CranioCatch, Eskisehir, Turkey).

**Figure 4:**
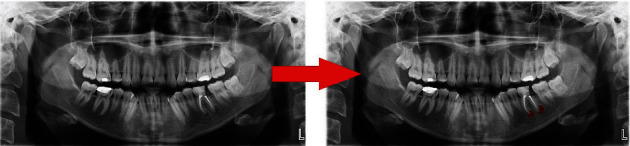
Automatically apical lesion segmentation using AI model (CranioCatch, Eskisehir, Turkey).

**Figure 5:**
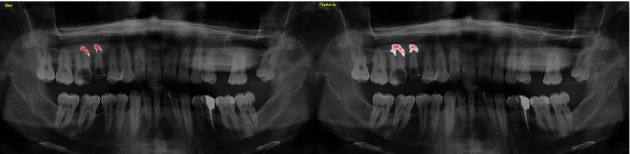
An example real-prediction image comparison.

The AI model segmented 63 apical lesions on 47 radiographs in the test data set (True Positives) (Figures 3–5).

Twelve apical lesions were not detected (False Negatives). In 5 cases without apical lesions, lesions were nevertheless segmented by the AI model (False Positives) (Table 1).

The sensitivity, precision, and F1-score values at 70% IoU value were 0.92, 0.84, and 0.88, respectively (Table 2).

### 3.2. Discussion

AI has rapidly improved the interpretation of medical and dental images, including via the application of deep learning models and CNNs [28, 29]. Deep learning has been developing rapidly thus recently attracting considerable attention [28–34]. The deep CNN architecture appears to be the most used deep learning approach. This is most likely due to its effective self-learning models and high computing capacity, which provide superior classification, detection, and quantitative performance based on imaging data [28–35]. CNNs have been used in dentistry for cephalometric landmark detection, dental structure segmentation, tooth classification, and apical lesion detection [36–39].

Tuzoff et al. presented a novel CNN algorithm for automatic tooth detection and numbering on panoramic radiographs. They found the sensitivity and specificity value of tooth numbering as 0.9893 and 0.9997, respectively. The findings showed the ability of current CNN architectures for automatic dental radiographic interpretation and diagnosis on panoramic radiographs [25]. Chen et al. detected and numbered teeth in dental periapical films using faster region proposal CNN networks (faster R-CNN). Faster R-CNN performed unusually well for tooth detection and localization, showing good precision and recall and overall performance like that of a younger dentist [24]. Miki et al. assessed the utility of deep CNN for classifying teeth based on dental CBCT images; the accuracy was 91.0%. The system rapidly and automatically produces diagrams for forensic recognition [38]. Two previous studies investigated the utility of AI systems for detecting periapical lesions. Ekert et al. investigated the capability of deep CNN algorithm to detect apical lesions on dental panoramic radiographs. CNNs detected the lesions despite the small number of data sets [6]. Orhan et al. [39] compared the diagnostic ability of a deep CNN algorithm to that of volume measurements based on CBCT images in the context of periapical pathology. The rate of detection of periapical lesions of the CNN model was 92.8%, and the volumetric and manual segmentation measurements were similar [39]. Endres et al. [40] created a model using 2902 deidentified panoramic radiographs. The presence of periapical radiolucencies on panoramic radiographs was evaluated by 24 oral and maxillofacial surgeons. They show that the deep learning algorithm has better success than 14 of 24 oral and maxillofacial surgeons. The success metrics for this model were as follows: the precision of 0.60 and an F1 score of 0.58 corresponding to a positive predictive value of 0.67 and True Positive rate of 0.51. Setzer et al. performed a study to use a deep learning proposal using U-Net architecture for the automatic segmentation of periapical lesions on CBCT images [41]. Segmentation of lesion accuracy was found as 0.93 with a specificity of 0.88, a positive predictive value of 0.87, and a negative predictive value of 0.93. They concluded that the DL algorithm trained in a limited CBCT images presented wonderful results in lesion detection accuracy. In the presented study, we created a segmentation model with PyTorch U-Net AI architecture on panoramic radiograph. It segmented 63 apical lesions on 47 radiographs in the test data set. Twelve apical lesions were not detected. In 5 cases without apical lesions, lesions were nevertheless segmented by the AI model. The sensitivity, precision, and F1-score values at 70% IoU value were 0.92, 0.84, and 0.88, respectively. Our results showed that AI deep learning algorithms can have service ability in the clinical dental setting. However, the present study had some limitations. Only one radiography machine and standard parameters were used to image acquisitions. Besides, study groups included all size of periapical images. The external test group was not used to assess the model's success. We used the U-Net algorithm to model development, only. Future studies should be used using larger study samples and images taken from different radiography equipment. Comparative experiments should be planned to use different CNN algorithms, and AI model performance should be compared to different human observers which have different level professional experiences.

## 4. Conclusions

Deep learning AI models enable the evaluation of periapical pathology based on panoramic radiographs. The application of AI for apical lesion detection and segmentation can reduce the burden on clinicians.

## Figures and Tables

**Table 1:** The number of segmented apical lesions with AI model (CranioCatch, Eskisehir, Turkey).

Metrics	Number
True Positives (TP)	63
False Negatives (FN)	12
False Positives (FP)	5

**Table 2:** The prediction performance measurement of the AI model (CranioCatch, Eskisehir, Turkey).

Measure	Value	Derivations
Sensitivity (recall)	0.92	TP/(TP + FN)
Precision	0.84	TP/(TP + FP)
F1 score	0.88	2TP/(2TP + FP + FN)
IoU value	0.79	TP/(TP + FP + FN)
Dice coefficient	0.88	2TP/(2TP + FP + FN)

## Data Availability

The data used to support the findings of this study are available from the corresponding author upon request.
